# Effect of Zn Addition on the Microstructure, Mechanical Properties, and Fracture Behavior of As-Cast Mg-Gd-Y-Zr Alloys

**DOI:** 10.3390/ma16072720

**Published:** 2023-03-29

**Authors:** Xiangsheng Xia, Echuan Yang

**Affiliations:** 1 Southwest Technology and Engineering Research Institute, Chongqing 400039, China; 2 School of Mechanical Engineering, Chongqing University of Technology, Chongqing 400054, China

**Keywords:** magnesium alloy, LPSO phase, microstructure, mechanical properties, fracture behavior

## Abstract

The microstructure and mechanical properties of semi-continuous casting Mg-Gd-Y-Zr magnesium alloys with different Zn contents were studied in this paper. The results showed that an increase in Zn content resulted in gradual refinement of the grains and a gradual increase in the volume fraction of the second phase. At a Zn content of 0.7 wt%, the microstructure was mainly composed of the α-Mg matrix and the Mg5(GdY) and long-period stacking order (LPSO) phases. An increase in the Zn content lowered the volume fraction of the Mg5(GdY) phase and increased the volume fraction of the LPSO phase. At a Zn content of 3.3 wt%, the microstructure was mainly composed of the α-Mg matrix and the LPSO phase. Among these alloys, the alloy without Zn addition showed an optimal ultimate tensile strength and yield strength of 229 MPa and 185 MPa, respectively, while the alloy with 3.3 wt% Zn showed an excellent elongation after fracture of 4.5%. The tensile fracture analysis indicated that the cracks of the alloy without Zn mainly originated at the trigeminal junction of the grain boundary, the cracks of the 0.7 wt% Zn and 1.5 wt% Zn alloy mainly originated at the interface of the Mg/lamellar LPSO phase, and the cracks of the 3.3 wt% Zn alloy mainly originated at the bulk LPSO phase of the grain boundary and then propagated along the bulk LPSO phase.

## 1. Introduction

At present, energy and environmental issues have become a worldwide focus; the implementation of a lightweight design has been suggested as an effective way to minimize these issues. Because of its low density and high strength ratio, magnesium alloys have broad application prospects in the fields of automobile, electronics, aerospace, and national defense [1,2,3,4]. Compared with medium strength aluminum alloys, commercial AZ31, ZK60, and AZ80 magnesium alloys possess a low strength and poor thermal stability, which limit their widespread application. In order to promote their applicability across a variety of fields, researchers have focused on developing high-strength magnesium alloys [5,6,7,8]. In general, the rare earth (RE) element Gd has been widely used in the alloying of magnesium alloys, and Mg-Gd magnesium alloys are characterized as typical Mg-RE magnesium alloys [9,10,11]. In order to achieve higher mechanical properties, other RE elements, such as Y, Nd, and Ce, are usually added to Mg-Gd magnesium alloys. In addition, Zn is often added to the Mg-RE alloy to enhance its aging strengthening effect [12,13,14,15,16,17]. Homma et al. [14] reported a Mg-10Gd-5.7Y-1.6Zn-0.6Zr (wt%) alloy with a tensile strength and yield strength of approximately 542 MPa and 473 MPa, respectively. Xu et al. [15] examined an Mg-8.2Gd-3.8Y-1.0Zn-0.4Zr alloy, which exhibited a tensile strength, yield strength, and elongation of 517 MPa, 426 MPa, and 4.5%, respectively, after high strain hot rolling and aging. Wang et al. [16] reported that the tensile strength, yield strength, and elongation of an aged Mg-9.2Gd-3.3Y-1.2Zn-0.9Mn alloy with a large amount of long-period stacking order (LPSO) and β’ phases reached 525 MPa, 420 MPa, and 6.3%, respectively.

In recent years, research has mainly focused on the effect of plastic deformation and heat treatment on the microstructure and mechanical properties of Mg-Gd-Y-Zn-Zr alloys [18,19,20,21]. The effect of Zn addition on the microstructure and mechanical properties of as-cast Mg-Gd-Y-Zr alloys has been minimally reported. This paper examined the microstructures and mechanical properties of as-cast Mg-8.0Gd-4.5Y-0.5Zr alloys at various Zn addition levels, after which their fracture behaviors were discussed. The results of this study may provide a theoretical basis for the alloying design of Mg-Gd-Y-Zn-Zr alloys.

## 2. Materials and Methods

The nominal compositions of the studied alloys were Mg-8.5Gd-4.5Y-xZn-0.4Zr (x = 0, 0.7, 1.5, and 3 wt%). The ingots of the four as-cast alloys were prepared in a frequency induction melting furnace. The pure Mg and pure Zn were completely melted in a crucible, to which Mg-30 wt% Y/Gd master alloys were added when a melting temperature of 780 °C was reached. The melting temperature was subsequently increased to 800 °C, after which the Mg-30 wt% Zr alloy was added into the melt. The melt was then fully stirred by Ar gas for 20 min and then kept static for 60 min. The semi-continuous casting was performed to obtain ingots with a diameter of Φ162 mm and a length of 4000 mm at a temperature of 680 °C. Table 1 lists the chemical compositions of the alloys examined by inductivity coupled plasma-atomic emission spectroscopy (ICP-AES). The Zn contents of the samples, from low to high levels, were abbreviated as 0Zn, 0.7Zn, 1.5Zn, and 3.3Zn alloys.

The microstructures of the alloys were observed by optical microscopy (OM, Carl Zeiss Axiovert 2000, Oberkochen, Germany), scanning electron microscopy (SEM, Carl Zeiss Evo 18), and transmission electron microscopy (TEM, FEI Tecnai F20). The OM and SEM surface observations were etched using a solution of 4% HNO3 with ethanol. The TEM observation samples were twin-jet electro-polished in a solution of 15 mL perchloric acid and 285 mL ethanol at −30 °C and 0.01 A. The phase composition of the alloys was analyzed using a D8 discover X-ray diffractometer. The cylinder sample used for the X-ray analysis was machined as casting rods with a diameter of 15mm. The mechanical properties were analyzed using an MTS material testing machine, and the stretching samples were manufactured according to the standard of GB/T 16865-2013.

## 3. Results

### 3.1. Microstructure

**Figure 1:**
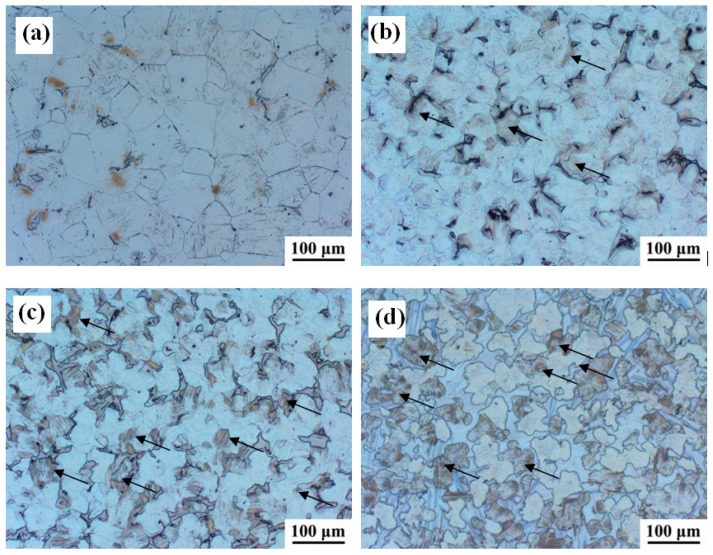
OM images of the experimental alloys: (**a**) 0Zn, (**b**) 0.7Zn, (**c**) 1.5Zn, and (**d**) 3.3Zn.

Figure 1 shows the OM images of the experimental alloys with different Zn contents. The four alloys were mainly composed of an α-Mg matrix and exhibited second phase crystallization at the grain boundaries, wherein the second phase was positively correlated to the Zn content. For the 0Zn alloy, a small number of discontinuous second phases were observed at the grain boundaries, especially at the triangular grain boundaries. With the increase in Zn content, the volume fraction of the second phase gradually increased, and the continuous network second phase was formed, as illustrated in Figure 1d. Furthermore, the grain sizes of the alloys with Zn addition were relatively smaller than that of the Zn-free alloy; as the Zn content increased from 0.7 wt% to 3.3 wt%, the average grain size of alloy decreased from 60.3 to 46.6 µm, indicating that the addition of Zn to the Mg-Gd-Y-Zr alloy produced somewhat finer grains. Similar results were also reported in Mg-8Gd-xZn-0.4Zr alloys [22]. Compared with that of the 0Zn alloy, some fuzzy second phases were observed inside some grains in the 0.7Zn, 1.5Zn, and 3.3Zn alloys, besides the second phase at the grain boundaries, as shown by the arrow in Figure 1b–d. In addition, these fuzzy second phases gradually increased with the increase in Zn content.

**Figure 2:**
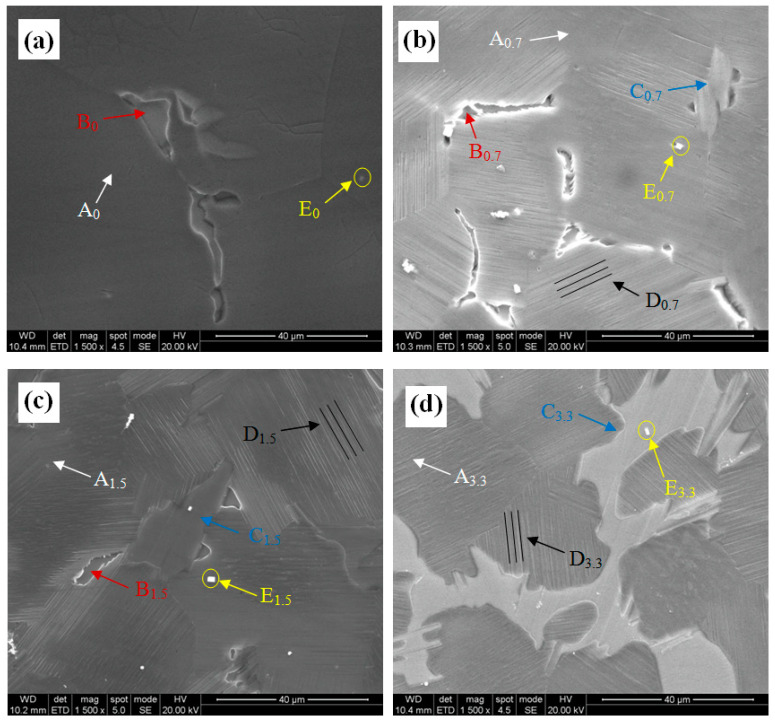
SEM images of the experimental alloys: (**a**) 0Zn, (**b**) 0.7Zn, (**c**) 1.5Zn, and (**d**) 3.3Zn.

Figure 2 shows the SEM images of the experimental alloys at different Zn contents. For the 0Zn alloy, the microstructure was mainly composed of an α-Mg matrix (A0 point in Figure 2) and a second phase (B_0_ point in Figure 2), wherein the second phase exhibited eutectic phases. For the alloys with Zn addition, the second phases were composed of two phases, the first one was the eutectic phase (B point in Figure 2b–d) and the second one was the raised bulk phase (C point in Figure 2b–d). As the Zn content was increased, the volume fraction of the raised bulk phase was increased, while the volume fraction of the eutectic phase was decreased. In addition, platelets (D point in Figure 2) and cuboid phases (E point in Figure 2) were observed. The platelets were parallel to each other in the same grain.

**Figure 3:**
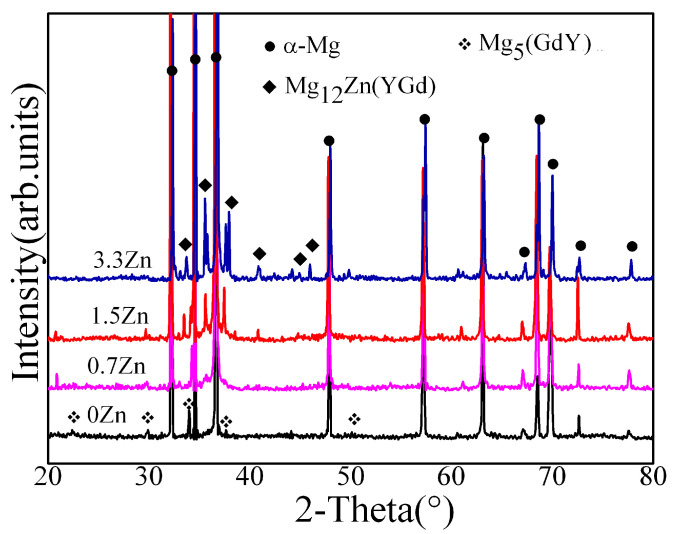
XRD patterns of the experimental alloys.

To reveal the crystal structures of the second phases, XRD characterization was conducted, and the XRD patterns of the experimental alloys with different Zn contents are shown in Figure 3. The XRD pattern of the 0Zn alloy mainly showed the diffraction peaks of the α-Mg matrix and the Mg_5_(GdY) phase, which correspond to A and B in Figure 2, respectively. The XRD pattern of the 0.7Zn alloy mainly showed the diffraction peaks of the α-Mg matrix, while the Mg_5_(GdY) phase, the Mg12Zn(YGd) phase, and the Mg_5_(GdY) phase exhibited smaller diffraction peaks than the 0Zn alloy. An increase in the Zn content (1.5 wt%) resulted in a further decrease in the Mg_5_(GdY) phase diffraction peak intensity, while that of the Mg_12_Zn(YGd) phase increased. The diffraction peaks of the α-Mg matrix and the Mg_12_Zn(YGd) phase were mainly found on the XRD pattern of the 3.3 Zn alloy, although the diffraction peaks of the Mg_5_(GdY) phase disappeared. Interestingly, the Mg_12_Zn(YGd) phase was determined to be an LPSO phase [23]. Therefore, the Zn addition promoted the formation of the LPSO phase, but suppressed the formation of the Mg_5_(GdY) phase. Combined with the results of the SEM images and XRD patterns, the second phases B and C in Figure 2 were determined to be Mg_5_(GdY) and LPSO phases, respectively.

**Figure 4:**
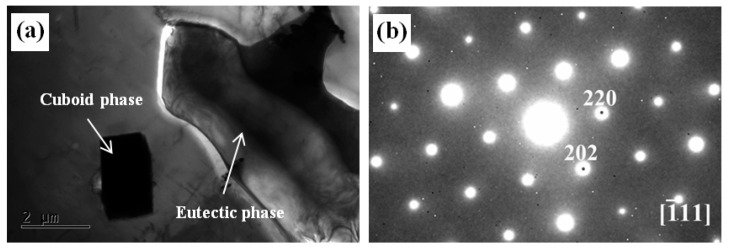
TEM analysis results of the as-cast 0Zn alloy: (**a**) bright field image and (**b**) selected area electron diffraction of the eutectic phase.

In order to further compare and reveal the similarities and differences of the second phases in the alloys with and without Zn addition, the microstructures of the 0Zn and 1.5Zn alloys were characterized by TEM. Figure 4 shows the TEM bright-field (BF) image and the corresponding selected area electron diffraction (SAED) pattern of the 0Zn alloy. The cuboid phase and the eutectic phase were observed near the grain boundary. The electron beam could not penetrate the cuboid phase, and the corresponding SAED pattern was not obtained. The EDS results indicate that the cuboid phase had a chemical composition of 8.34at% Mg–28.73 at% Gd–62.92at% Y, which is a RE-rich phase. The SAED pattern suggests that the Mg5(GdY) phase (FCC, FM, a = 2.23 nm) was a eutectic phase.

**Figure 5:**
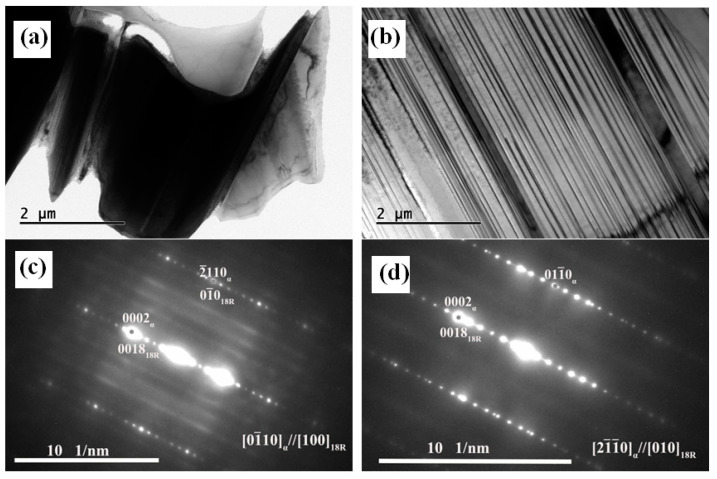
TEM analysis results of the as-cast 1.5Zn alloy: (**a**) bright field image of the block phase, (**b**) bright field image of the lamellar structure, (**c**) selected area electron diffraction of the block phase, and (**d**) selected area electron diffraction of the lamellar structure.

Figure 5 shows the TEM analysis results of the 1.5Zn alloy. As shown in Figure 5a and Figure 5c, the block phase had an 18R LPSO phase. In addition to the bulk LPSO phase at the grain boundary, some lamellar structures were distributed in parallel along one direction near the grain boundary. According to the diffraction pattern of the lamellar structure, the lamellar structure was also an 18R LPSO phase.

### 3.2. Mechanical Properties

**Figure 6:**
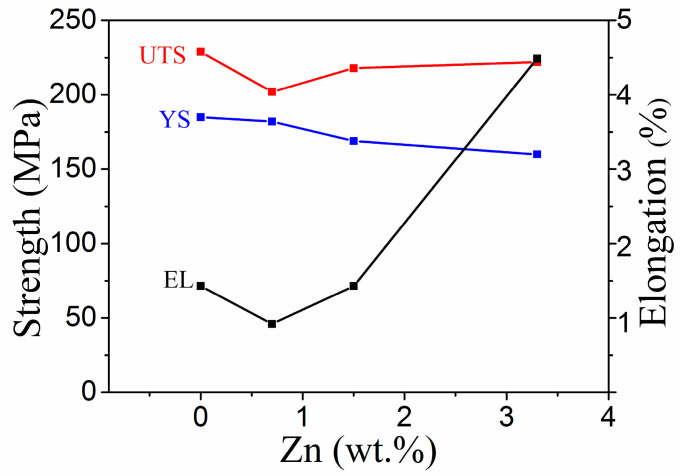
Variation in the mechanical properties of the alloy at varying Zn contents.

Figure 6 shows the changes in the tensile strength, yield strength, and elongation of the alloy with Zn content. An increase in the Zn content first resulted in a decrease in the tensile strength of the alloy, followed by an increase, although the range of the changes was not large. The yield strength gradually decreased with an increase in the Zn content, while the elongation after fracture first decreased and then increased with an increase in the Zn content, especially when the Zn content exceeded 1.5 wt%. The 0Zn alloy exhibited a relatively high strength (UTS of 211 MPa and YS of 164 MPa) but relatively low ductility (EL of 1.2%). The 0.7Zn alloy exhibited a reduced UTS of 201 MPa, YS of 146 MPa, and EL of 1.1%. For the 1.5Zn alloy, an improved UTS of 218 MPa and EL of 2.0% were observed, while YS was slightly decreased. As the Zn content increased to 3.3 wt%, the YS significantly decreased by ~44 MPa (from 164 MPa to 120 MPa), while the EL increased from 1.2% to 4.1% compared with that of the 0Zn alloy. Generally speaking, the 0Zn alloy exhibited the highest strengths—a tensile strength and yield strength of 229 MPa and 185 MPa, respectively—while the 3.3 wt% Zn alloy showed the highest elongation after fracture (4.5%).

### 3.3. Fracture Behaviors

**Figure 7:**
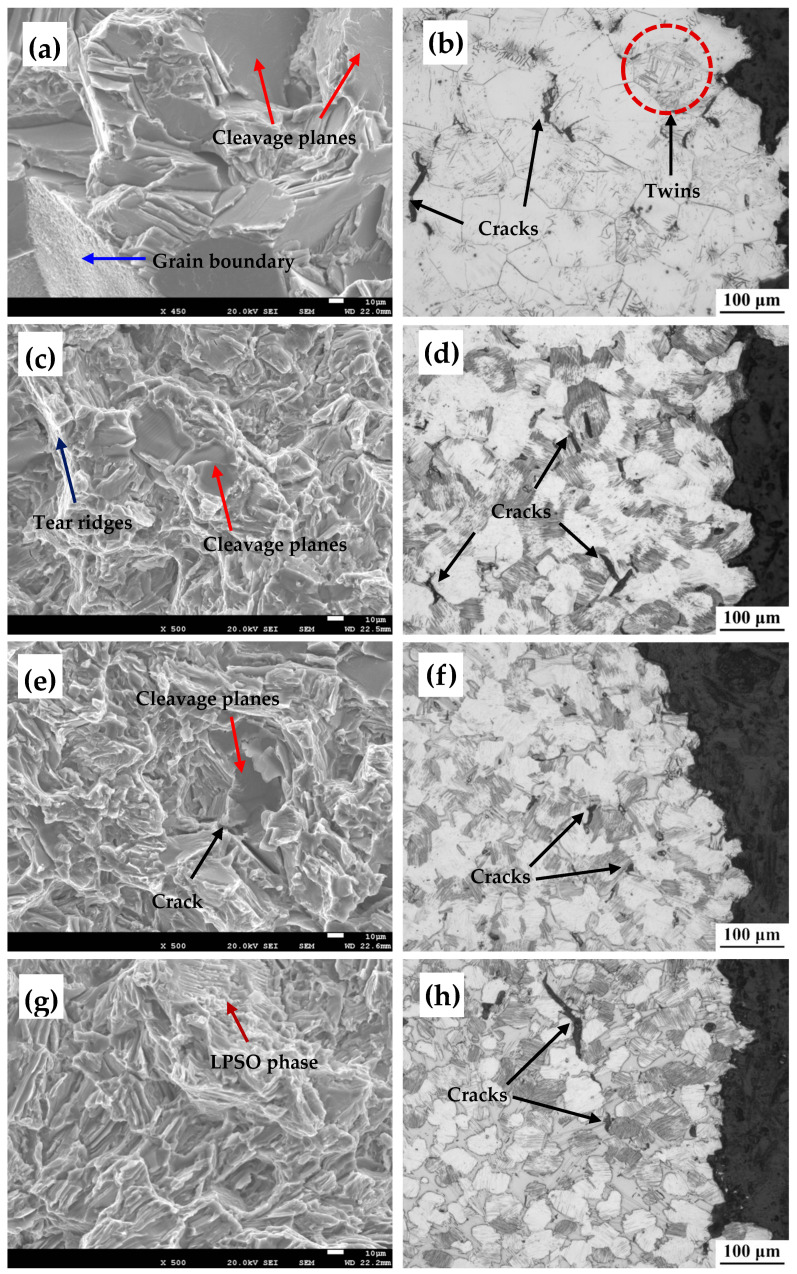
Tensile fracture surface and microstructure near the fracture surface of the alloy: (**a**,**b**) 0Zn, (**c**,**d**) 0.7Zn, (**e**,**f**) 1.5Zn, and (**g**,**h**) 3.3Zn.

Figure 7 shows the microstructures in and around the tensile fracture surfaces of the as-cast 0Zn, 0.7Zn, 1.5Zn, and 3.3Zn alloys. Figure 7a shows that the fracture morphology of the 0Zn alloy exhibited co-existing transgranular and intergranular fractures, which resulted in brittle fracture. Cracks were mainly observed at the grain boundaries in the microstructure analysis near the fracture surface, especially at the junction of the grain boundaries, mainly due to the presence of minimal slip systems at room temperature and stress concentration at the grain boundaries. These were largely observed at the trigeminal node of the grain boundaries, where cracks first occurred, such that the formation of cracks resulted in gradual expansion along the grain boundaries or grains. Because of the different orientations of the grains, twins were also observed in some grains, as shown in Figure 7b. After adding 0.7 wt% Zn, the fracture mode also exhibited co-existing transgranular and intergranular phases. The microstructure analysis near the fracture surface showed that the cracks were mainly initiated at the grain boundary and within the grain, and the cracks within the grain were mainly parallel to the lamellar LPSO phase. With the Zn content increasing to 1.5 wt%, the alloy did not exhibit any significant changes in the fracture mode. However, when the Zn content increased to 3.3 wt%, the fracture morphology transformed to intergranular fracture, and the crack was mainly exhibited in the bulk LPSO phase at the grain boundary. In contrast, the volume fraction of the bulk LPSO phase at the grain boundary increased to a level that allowed for gradual crack propagation along the bulk LPSO phase.

## 4. Discussions

### 4.1. Effect of Zn Content on the Microstructure of the As-Cast Alloy

**Figure 8:**
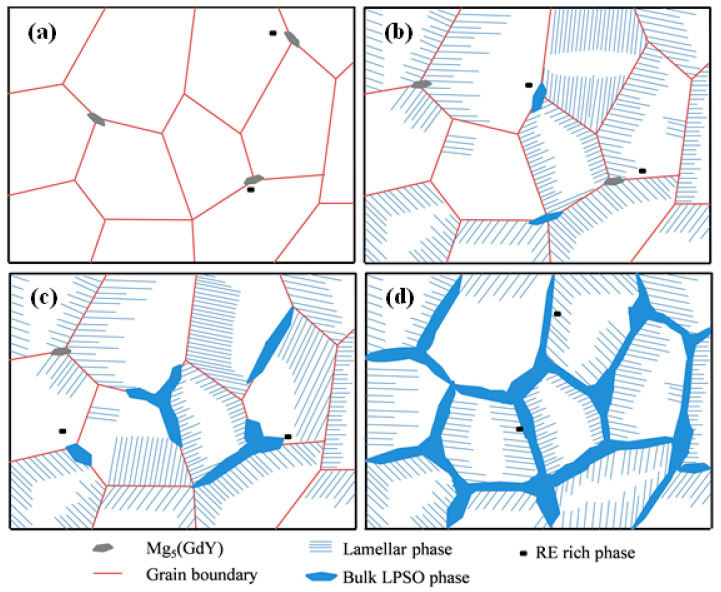
Effect of the Zn contents on the microstructures of the alloys: (**a**) 0Zn, (**b**) 0.7Zn, (**c**) 1.5Zn, and (**d**) 3.3Zn alloys.

According to the analysis in Section 3.1, the number and type of the second phase were dependent on the Zn content, the influence of which is shown in Figure 8. When the Zn content was not added, only the Mg_5_(GdY) phase was observed at the grain boundary. After adding 0.7 wt% Zn, the grain boundary contained the Mg_5_(GdY) phase and the bulk LPSO phase, and the lamellar LPSO phase was observed within grain. An increase in the Zn content to 1.5 wt% resulted in a decrease in the number of eutectic Mg_5_(GdY) phases, although the number of LPSO phases increased. When the Zn content further increased to 3.3 wt%, only the LPSO phase was present—the eutectic Mg_5_(GdY) phase was not observed. These results indicate that the number of LPSO phases in the alloy was significantly dependent on the Zn content. The atomic radius of RE(Gd/Y) was larger than that of Mg, while the atomic radius of Zn was smaller than that of Mg. When RE and Zn segregated, the lattice distortion caused by RE was weakened, which promoted the segregation of RE and Zn. In addition, when the RE and Zn elements were able to co-exist, the stacking fault energy was significantly reduced, which promoted the formation of stacking faults [24]. Therefore, an increase in the Zn content resulted in the participation of more Zn atoms in the formation of the LPSO phase, resulting in an increase in the number of LPSO phases.

An increase in the Zn content resulted in a decrease in the number of eutectic Mg_5_(GdY) phases and an increase in the LPSO phase. In addition, only the LPSO phase was observed in the 3.3Zn alloy, indicating that the LPSO phase was formed before the Mg_5_(GdY) phase during solidification, and Zn and RE were continuously enriched at the interface of the solidification front. When the conditions were met, the LPSO phase was formed and the alloying elements Zn and RE were consumed. At a large enough Zn element content, the RE element was consumed in large quantities, which inhibited the formation of the Mg_5_(GdY) phase.

### 4.2. Effect of the LPSO Phase on the Fracture Mechanism

**Figure 9:**
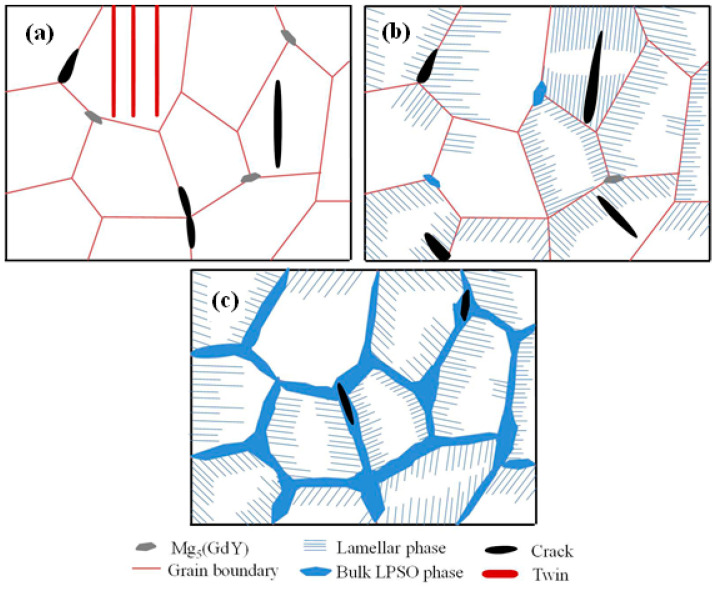
Schematic diagram of fracture of the as-cast alloys: (**a**) 0Zn, (**b**) 0.7Zn/1.5Zn, and (**c**) 3.3Zn alloys.

From the analysis of the above section, the addition of different Zn contents resulted in obvious differences in the as-cast microstructure of the alloy. The main difference was observed in the morphology and distribution of the LPSO phase, which also resulted in significant changes in the fracture mechanism of the alloy. Figure 9 shows the schematic diagram of fracture of the as-cast 0Zn, 0.7/1.5Zn, and 3.3Zn alloys. When Zn was not added, twins were observed in some grains, and cracks were first initiated at the trigeminal node of the grain boundary, where the strong stress concentration occurred (Figure 9a). When 0.7/1.5 wt% Zn was added, many lamellar LPSO phases were observed and cracks were mainly initiated at the interface of the Mg/lamellar LPSO phases (Figure 9b); a similar result was observed in the Mg-9.5Gd-4Y-2.2Zn-0.5Zr alloy [25]. Following the addition of 3.3 wt% Zn, the bulk LPSO phase was distributed in a network along the grain boundary. In addition, cracks were mainly initiated in the bulk LPSO phase at the grain boundary and then propagated along the bulk LPSO phase, thereby producing intergranular fracture characteristics (Figure 9c). Crack sources were easily formed at the interface of the Mg/bulk LPSO phase and in the bulk LPSO phase. The formed cracks were propagated along the bulk LPSO phase/bulk LPSO phase, indicating that the LPSO phase had less resistance to crack propagation.

## 5. Conclusions

In this paper, the microstructures and mechanical properties of as-cast Mg-8.5Gd-4.5Y-0.4Zr alloys at various levels of Zn content additions were studied, after which their fracture behaviors were discussed. The main conclusions are summarized as follows:

(1) An increase in Zn content resulted in a gradual refinement of the grain size; as the Zn content increased from 0.7 wt% to 3.3 wt%, the average grain size of alloy decreased from 60.3 to 46.6 µm. In addition, the volume fraction of the second phase gradually was increased.
(2) The microstructure of the 0Zn alloy mainly consisted of an α-Mg matrix and the Mg5(GdY) phase; the microstructure of the 0.7Zn alloy mainly consisted of an α-Mg matrix, the Mg5(GdY) phase, and the LPSO phase. An increase in the Zn content resulted in a decrease in the Mg5(GdY) phase volume fraction and an increase in the LPSO phase volume fraction. The microstructure of the 3.3Zn alloy mainly consisted of an α-Mg matrix and the LPSO phase.
(3) The alloy without any added Zn showed an optimal ultimate tensile strength and yield strength of 229 MPa and 185 MPa, respectively, while the alloy with 3.3 wt% Zn showed an excellent elongation of 4.5% after fracture.
(4) Cracks in the 0Zn alloy mainly originated at the trigeminal junction of the grain boundary, whereas cracks in the 0.7Zn and 1.5Zn alloys mainly originated at the interface of the Mg/lamellar LPSO phase. In comparison, cracks in the 3.3Zn alloy mainly originated at the bulk LPSO phase at the grain boundary and then propagated along the bulk LPSO phase.

## Figures and Tables

**Table 1:** Actual chemical compositions of the experimental alloys (wt.%).

Alloy	Gd	Y	Zn	Zr	Mg
0Zn	8.1	4.5	0.0	0.3	Bal.
0.7Zn	8.5	4.5	0.7	0.4	Bal.
1.5Zn	8.2	4.6	1.5	0.4	Bal.
3.3Zn	8.2	4.5	3.3	0.4	Bal.

## Data Availability

Not applicable.
